# A novel self-report instrument (APPA) to measure psychotic symptoms – development and content validity

**DOI:** 10.1192/j.eurpsy.2025.719

**Published:** 2025-08-26

**Authors:** B. E. Sportel, A. Vegter, T. van de Giessen, G. H. M. Pijnenborg, G. D. van Rijsbergen

**Affiliations:** 1 Psychotic disorders, GGZ Drenthe Mental Health Institute, Assen; 2 Youth and Adolescent psychiatry, GGZ Drenthe Mental Health Institute, Hoogeveen; 3 Clinical and developmental neuropsychology, University of Groningen, Groningen, Netherlands

## Abstract

**Introduction:**

Various interviews are available to measure psychotic symptoms, including e.g. the Positive And Negative Syndrome Scale (PANSS) when a psychotic disorder is suspected or the Comprehensive Assessment of At Risk Mental States (CAARMS) when there is a suspicion of an increased risk of developing a first psychosis. Conducting these interviews is time-consuming (60-90 minutes) for both patient and professional, and requires a trained interviewer. To our knowledge, no self-report questionnaire is available to measure the degree of psychotic symptoms, while such an instrument is desirable to measure e.g. treatment effects.

**Objectives:**

The aim of the current research: 1. to use gold standard interviews that measure psychosis spectrum problems to develop a self-report questionnaire based on DSM-V, and 2. to examine the content validity of the newly developed instrument.

**Methods:**

Based on the instruments PANSS, CAARMS and SCID-S (Structured Clinical Interview for DSM disorders) the first version of the Asser Psychotische Klachtenlijst (Asser Psychotic Phenomenology Assessment; APPA) was constructed. The APPA assesses symptoms over a three-month time period. To assess content validity, interviews took place with the target population and MH care professionals, followed by alterations based on the results.

**Results:**

Interviews were held with six patients (women: 2; age range: 19-52) with a psychotic disorder, within the first five years after diagnosis. Patients were positive about the questionnaire, thought it was comprehensible and gave a good reflection of their psychotic problems. Furthermore, they thought the APPA had clear instructions and a good scaling (5-point-Likertscale). Two out of the 36 questions were considered more difficult to comprehend. Eight MH care professionals (one nurse, one nurse practitioner, two psychiatrists, and four psychologists) were interviewed about the APPA. They considered the instrument to be clear and complete with regard to psychotic symptoms, with clear instructions and good scaling. In their opinion two questions were vague, 14 questions contained ambiguous words and five questions were formulated in a complicated way. Based on the findings of the target population and professionals, several questions were adjusted, or reformulated into multiple questions, resulting in a 41-item questionnaire on a 5-point-Likertscale.

**Conclusions:**

The APPA was found to be a comprehensible, clear instrument, giving a good reflection of psychotic problems according to the target population and professionals. The content validity of the APPA was found to be good. As a next step we are collecting data within the target population to establish reliability, this will be followed by a study measuring other psychometric properties, in which the APPA will also be compared to gold standard instruments. The APPA thus far seems a promising instrument for both research and daily practice.

**Disclosure of Interest:**

None Declared

